# A Review of Its Medical Aspects.—II

**Published:** 1909-03-06

**Authors:** 


### THE POOR-LAW COMMISSION'S REPORT.

### A REVIEW OF ITS MEDICAL ASPECTS.—II.

(*Continued from page* 572.)

General Conclusions and Proposals.

(a) *Influence of Sickness on Pauperism.*

There is an intimate connection between sickness and pauperism. During a given year in the Union of Nottingham over 40 per cent., and in the parish of Glasgow about 50 per cent., of the applications for relief were tabulated as due to sickness or ill-health. Of 4,000 cases of consumption in the wards of a Union infirmary Dr. Nathan Raw, of Liverpool, came to the conclusion that 60 per cent. were paupers because they were consumptives; 30 per cent. of the total paupers are sick. We estimate that one-half of the total cost of pauperism is swallowed up in direct dealing with sickness, and, allowing for the indirect results, it is probable that to the extent to which we can eliminate or diminish sickness among the poor we shall eliminate or diminish one-half the existing amount of pauperism.

(b) *Objections to Gratuitous Medical Assistance.*

In the first place, so far as the prevention of disease is concerned, the free medical service already exists. The sanitary authorities freely apply preventive measures, and freely cure or attempt to cure many classes of disease which are a danger to the community. Pauperism due to preventable diseases must have diminished, the death-rate due to such diseases having been materially reduced during the last 40 years. The classes of diseases now dealt with by the sanitary authorities might perhaps with advantage be extended, and, so far as the prevention of disease is concerned, the question of a free medical service is confined to considering the amount of such extension.

So far as the cure of disease is concerned, the disadvantages of a free medical service outweigh its advantages, and we are confident that the greater part of these advantages are attainable by other measures. It would be impossible to confine it to the very poor, or even the poor. What the State provides should be the best possible, and, even with the present deterrents, the improvements in indoor medical relief have in 16 years doubled the number of ordinary able-bodied inmates who come for indoor relief in sickness. Also an increasingly higher class of society are availing themselves of these advantages. Either the comparatively well-to-do would crowd out the very poor, or the State would have to treat gratuitously practically the whole population. Further, it would inevitably tend to kill all the existing voluntary organisations for medically assisting the sick poor, and the practice of the majority of private medical practitioners would be gone. Finally, the cost would be prohibitive. Any method by which medical assistance is made more accessible to and more readily obtainable by the working classes should be such as not to diminish, but to encourage and stimulate, a feeling of independence and self-maintenance.

(c) *Objections to Transfer of Medical Assistance to Sanitary Authorities.*

It has been suggested that the administration of medical assistance should be handed over to the sanitary authority. We think the proposed transfer would be inconsistent with any but a gratuitous system. The sanitary authority has no existing staff for adequate inquiry as to the means of a patient; the Poor Law has. The sanitary authorities working in the interests of the community as a whole have tried to make their services attractive to all classes, and rightly or wrongly have allowed to fall into desuetude the power of recovering the cost of treatment in their hospitals, which, except in London, they legally possess.

There are over 1,800 sanitary authorities, and only 643 Poor-law Unions; the main contention of our report is that there is in every branch of the Poor Law a crying need of a larger area for purposes both of administration and classification.

As medical treatment at the present time is largely a question of dieting the patients, the sanitary authorities would have to extend their functions to the provision of maintenance. Two relief authorities would be created where only one exists at present. Further, many sanitary authorities, especially in rural districts, have not satisfactorily discharged their primary duties.

(d) *The Limits of the Practicable in the Reform of Medical Assistance.*

All methods for the prevention and cure of disease which have been generally approved by science and are reasonably accessible to the citizen of average means shall also be obtainable by his poorer neighbour upon payment of a contribution according to his means, and shall be made available for the very poor without any payment at all.

(e) *Analysis of Defects of Present System and Remedies Indicated.*

1. *Defects in Quality; and Remedies.*—When we consider the enormous improvements which have been made in the last 30 years, a great debt of acknowledgment is due, not only to the Local Government Board, but also to the local authorities. It is, however, clear from the report of our medical investigator and from our evidence that there still exist blemishes which cannot be justified in the service of both indoor and outdoor medical assistance. Their existence points in no uncertain manner to the need of more effective central and local medical supervision and control over the whole field. The staff of central inspectors should be increased, and the increase of medical control by the local authority should be attained by associating with that authority the assistance of some independent medical men. We recommend that the Public Assistance Authority should appoint from among their number a Medical Assistance Committee, to which shall be added representatives of various organisations more fully described later on. This committee should make a thorough survey of existing arrangements, study the needs of the locality, and suggest a complete system of medical assistance and inspection.

2. *Defects in Extent; and Remedies.*—At present a number of cases, by whom, in our opinion, it ought to be used, are not reached. This is chiefly due either to the physical inaccessibility of the service or to the mental aloofness of the people. But these causes of present evils can be eradicated through the machinery of a larger authority with special knowledge of the medical requirements and facilities of its area. The right degree of attractiveness and deterrence for the right class of people can best be decided by an authority which, while possessing the economic experience of the Poor-law authority, shall also possess experience of the medical habits of the people. The new Medical Assistance Committee should be associated with representatives of provident medical societies, and such societies should be officially encouraged.

3. *Defects from Overlapping; and Remedies.*—The dangers of overlapping are (*a*) a class of sick poor may be unprovided for—*e.g.* consumptives, because each authority thinks it is the duty of another authority to make provision; (*b*) one authority may be proceeding on principles directly subversive of those upon which the other authority acts; (*c*) the possible waste of money sorely needed in other directions. We recommend that the Medical Assistance Committee should have on its councils representatives of the other authorities and organisations engaged in the medical assistance of the poor.

4. *Medical Assistance as a Cause of Pauperism.*—We have been unable to find sufficient statistical basis for the view that medical relief, by reason of its association with the Poor Law, causes persons to become either a temporary or permanent burden on the rates after their sickness has been cured; but relieving officers are almost to a man convinced of this. It is probably not the medical relief but the sickness and its disabilities which cause the persons to come on the rates for maintenance. Any unnecessary pauperism thus caused is rather the result of lax administration. We find no reason for dissociating medical relief from the work of Public Assistance Authorities.

5. *Deterrence of Medical Assistance; and Remedies.*—It is in the interests of the community that the applicant should first be treated, and his economic position inquired into afterwards. The present method tends to deter people from seeking medical assistance when they properly should do. The association of sick persons in general workhouses with ordinary paupers deters many seeking indoor relief. This could be avoided by adopting wider areas for purposes of classification by institution, and thereby dissociating as far as possible medical institutions of the Public Assistance Authority from institutions dealing with classes other than the sick. The clearer definition of what comes under the head of medical relief is necessary to carry out the real intention of the Act of 1885 (Medical Relief Disqualification Removal). The investigation after treatment being by a different body to that which dealt with poor relief proper would decrease the reluctance of the poor to resort to it.

6. *The Proper Objects of Medical Assistance and Necessary Safeguards.*—The present system is deterrent to those who have a dislike to the Poor Law; while it is unduly attractive to those who like getting something for nothing. Medical assistance, while not being deterrent, should be such as to encourage independent provision against sickness wherever possible. The need for deterrence disappears if an application for medical assistance can be made the introduction to a provident dispensary. We recommend the following inducements to self-provision against sickness.

1. The power of recovering the cost or part cost should be retained and enforced where the recipient could have afforded to become a member of a provident dispensary. Such cost should be recoverable by instalments or remitted in whole or part, upon proof of the recipient having joined a provident dispensary.

2. The power of the committee to make investigation into means must be maintained, but it might be omitted in cases of members of provident dispensaries, provided a proper wage limit for membership were established and maintained.

There are difficulties in organising public medical assistance on a provident basis. (*a*) Largely, no doubt, owing to competition, medical men have contracted to give their services to the friendly societies at a lower rate than was desirable from any point of view. Some concordat on this question is of pressing importance. (*b*) The refusal of the medical profession to allow any canvassing for provident institutions with which its members are connected. (*c*) The refusal of the friendly societies to impose a wage limit for members paying the ordinary fee for medical attendance. Also, what part should the medical profession have in the management of the dispensaries and other provident institutions?

With regard to fees and wages limits, we think the British Medical Association should be requested to suggest a general scheme or scale of fees and wage limit to be applied by their local branches as local circumstances may suggest. Difficulties as to management will, we hope, be removed by the associated system we have suggested.

### Scheme of Reform.

The area of the future Public Assistance Authority will be the county and the county borough, with local committees working under it.

*General.*

1. That medical assistance should be reorganised on a provident basis.

2. That in certain cases power of compulsory removal to and detention in an institution should be given to the authorities, under proper safeguards.

3. That there should be systematic co-operation between the Public Assistance Authorities, the Sanitary Authorities, the Education Authorities, and the voluntary medical institutions, based on a clear definition of their respective functions.

4. That no disfranchisement should be attached to any form of medical assistance.

5. That the arrangements for indoor and outdoor medical assistance should be periodically inspected by medical inspectors on behalf of the Local Government Board.

*Functions of the Public Assistance Authority as to Medical Assistance.*

6. That in taking over the existing powers and duties of Boards of Guardians in regard to medical relief, the Public Assistance Authority shall undertake the following functions:—

(i.) To co-ordinate and, where necessary, supplement the medical institutions of the county or county borough, and to suggest methods of co-operation with the sanitary authorities and the authorities in charge of voluntary hospitals.

(ii.) To organise an outdoor and provident medical service easily accessible in all parts of the county or county borough, this service to include the provision of competent midwives.

(iii.) To develop an adequate nursing service, preferably in connection with voluntary nursing associations.

(iv.) To subscribe, when necessary, towards these purposes.

(v.) To arrange for adequate supervision of and report on the efficiency and adequacy of the medical institutions and medical service.

*Establishment of Medical Assistance Committees.*

7. That, to assist the Public Assistance Authority in carrying out the above functions, they shall appoint a committee from among their members, to which shall be added representatives of the Health Committees of the County Council or the County Borough Council, and of the local branch or branches of the British Medical Association. This committee shall be called the Medical Assistance Committee, and shall have power to co-opt representatives of local hospitals, nursing associations, dispensaries, and registered friendly societies.

8. That, where necessary, a local committee shall be appointed by each Public Assistance Committee for the purposes of the local administration of medical assistance.

9. That all or any of the functions defined in No. 6 may be referred by the Public Assistance Authority to the County or County Borough Medical Assistance Committee.

10. That the following shall be the lines of procedure for carrying out the functions mentioned in No. 6:—

(i.) To consider, in conjunction with each local Medical Assistance Committee, the needs of the county or county borough and the needs of each relief district in the way of hospital (including infirmary) accommodation.

(ii.) To reorganise existing Poor-law medical institutions throughout the county or county borough, with a view to making them more effective for different classes of patients. (This would be done in conjunction with the committees in charge of workhouses, schools, etc.)

(iii.) To organise schemes of co-operation between public assistance institutions and voluntary hospitals.

(iv.) To develop a system of paying and free cottage hospitals in conjunction with voluntary and endowed charities, these to include provision for confinement cases.

(v.) To arrange schemes of co-operation between hospital and provident dispensaries or outdoor medical service.

*Outdoor Medical Service.*

(i.) To require the local Medical Assistance Committee, in conjunction with the local doctor, to map out their district into convenient medical districts or "dispensary areas."

(ii.) To approach any free or provident medical institution, including private medical clubs, already existing in the district, and invite them to fall into line with the scheme (refusal not to check procedure).

(iii.) The local authority may subscribe to the provident dispensaries, subject to the sanction of the Local Government Board, and under the conditions mentioned in Section 10 of the Poor Law Act, 1879.

*Relation of the Poor Law to Provident Dispensaries.*

11. That where any applicant for medical assistance is not a member of a provident society, he shall apply to and at once be treated by a district medical officer.

12. That the relieving officer, or, as he will be called in future, the assistance officer, shall inquire into and report the case to the Public Assistance Committee, whose action shall be directed towards (*a*) recovery of cost; (*b*) strengthening of provident agencies.

13. That with a view to enlisting the services of all competent medical practitioners in the locality of a provident dispensary, the British Medical Association be requested to suggest a general scheme of fees and wage limit to be applied by their local branches as local circumstances may suggest.

14. That under such scheme all local medical practitioners consenting to come under it shall be on the list of dispensary doctors, and any applicant who is a member of the dispensary shall be entitled to select such practitioners as he may prefer.

15. That arrangements should be made, where necessary, for the reception of patients in voluntary and general hospitals by the payment of their cost or by some other agreement between the Public Assistance Committee and those in management of the hospital.

16. That any practitioner attached to a provident dispensary may, where the patient is a member of such dispensary and in need of institutional treatment, recommend him for admission to the local public assistance infirmary, and in certain cases, where arrangements have been made, to a voluntary or general hospital.

17. That so far as the patient is concerned, admission under such conditions shall be covered by the subscription to the provident dispensary.

18. That as regards certain cases in receipt of public assistance (such as the aged and widows with young children) the Public Assistance Committee may enroll all such cases as members of a provident dispensary by paying the necessary fees.

The Majority Report is signed as follows :—

| | |
|---|---|
| George Hamilton. | Charles S. Loch. |
| ✠ Denis Kelly. | J. Patten MacDougall. |
| H. A. Robinson. | Thomas Hancock Nunn. |
| S. B. Provis. | L. R. Phelps. |
| F. M. Bentham. | W. Smart. |
| (1) Arthur Downes. | Helen Bosanquet. |
| Thorn Gage Gardiner. | (2) Octavia Hill. |

(1) and (2) sign subject to reservations, some of which affect the question of medical relief.

Miss Octavia Hill considers the scheme of medical relief to be, in her opinion, unworkable, and, moreover, to open the door too widely to free medical relief. Dr. Downes' able Memorandum deals with both the Majority and Minority Reports, and an analysis of it will therefore appear at the end of the abstract of the latter.

(*To be continued.*)

